# A bioinformatics e-dating story: computational prediction and prioritization of receptor-ligand pairs

**DOI:** 10.1186/1471-2105-13-S18-A7

**Published:** 2012-12-14

**Authors:** Ernesto Iacucci, Léon-Charles Tranchevent, Dusan Popovic, Georgios A Pavlopoulos, Bart De Moor, Reinhard Schneider, Yves Moreau

**Affiliations:** 1 IBBT Future Health Department / ESAT-SCD, KU Leuven, Kasteelpark Arenberg 10, 3001, Heverlee-Leuven, Belgium; 2 Bioinformatics/Structural and Computational Biology, European Molecular Biology Laboratory, Meyerhofstrasse 1, 69117 Heidelberg, Germany; 3 Luxembourg Centre for Systems Biomedicine (LCSB), University of Luxembourg, Campus Limpertsberg, 162 A, Avenue de la Faïencerie, 1511 Luxembourg, Germany

## Background

Regulation of cellular events is initiated, often, via extracellular signaling when a circulating protein ligand interacts with one or more membrane-bound protein receptors. Identification of receptor-ligand pairs is thus an important and difficult task to address as this form of interaction is transient and not well studied. In order to address this problem, we collect the most readily available data from repositories (expression, domain, pathway, sequence, and text-based), and apply a high through-put analysis to this problem.

## Results

We have worked on the receptor-ligand pairing problem in three main studies. In our first study, using a LS-SVM classifier, we show that we are able to more aptly match members of the chemokine and tgfβ families than a previously published method [1]. Notably, we are able to achieve an increase in recall of 0.76 over the 0.44 for the matching of receptor-ligands in the tgfβ family. In our subsequent study, we benchmarked several machine learning techniques, and essayed several parameters, on the receptior-ligand interaction prediction task. We found that we could reach a balanced accuracy of 0.84. In our final work, we produce a publicly available database of our results with respect to a text-based in silico prediction workflow. The resulting database, contains several key findings, particularly predictions in the GPCR family with a balanced accuracy of 0.96.

## Conclusions

The receptor-ligand prediction task is an essential one, as the challenge of predicting such pairs is an important issue in wet-labs, biotech, and pharmaceutical companies. Through several studies, we have determined the most appropriate methodology to predict the receptor-ligand pairs and have made available high-quality predictions at our ReLianceDB website (http://homes.esat.kuleuven.be/~bioiuser/ReLianceDB), a tool to aid in performing effective and targeted research.
